# The Heparan Sulfate Binding Peptide in Tumor Progression of Triple-Negative Breast Cancer

**DOI:** 10.3389/fonc.2021.697626

**Published:** 2021-08-04

**Authors:** Carina Mucciolo Melo, Huawei Wang, Ken Fujimura, Jan Strnadel, Maria Cecília Zorél Meneghetti, Helena Bonciani Nader, Richard L. Klemke, Maria Aparecida Silva Pinhal

**Affiliations:** ^1^Department of Biochemistry/Molecular Biology, Universidade Federal de São Paulo, São Paulo, Brazil; ^2^Department of Biochemistry, Faculdade de Medicina do ABC, Santo André, Brazil; ^3^Department of Pathology, School of Medicine, University of California San Diego, La Jolla, CA, United States; ^4^Department of Molecular Medicine, Biomedical Center Martin, Jessenius Faculty of Medicine in Martin, Comenius University in Bratislava, Martin, Slovakia

**Keywords:** anti-angiogenic, breast neoplasia, glycosaminoglycans, phage display, breast cancer (BC)

## Abstract

Angiogenesis is the formation of new vessels from pre-existing vasculature. The heparan sulfate chains from endothelial cell proteoglycans interact with the major angiogenic factors, regulating blood vessels´ formation. Since the FDA´s first approval, anti-angiogenic therapy has shown tumor progression inhibition and increased patient survival. Previous work in our group has selected an HS-binding peptide using a phage display system. Therefore, we investigated the effect of the selected peptide in angiogenesis and tumor progression. The HS-binding peptide showed a higher affinity for heparin N-sulfated. The HS-binding peptide was able to inhibit the proliferation of human endothelial umbilical cord cells (HUVEC) by modulation of FGF-2. It was verified a significant decrease in the tube formation of human endothelial cells and capillary formation of mice aorta treated with HS-binding peptide. HS-binding peptide also inhibited the formation of sub-intestinal blood vessels in zebrafish embryos. Additionally, in zebrafish embryos, the tumor size decreased after treatment with HS-binding peptide.

## Introduction

Angiogenesis is forming new vessels from pre-existing vasculature during embryonic development and adult life (1).

Among the angiogenic factors that participate in neovascularization, we highlight the vascular endothelial growth factor (VEGF), hypoxia-induced factor (HIF), placental growth factor (PGF), fibroblast growth factor (FGF), angiopoietin-1 (Angp), platelet-derived growth factor (PDGF), tumor necrosis factor (TNF), interleukins (IL), epidermal growth factor (EGF), insulin-like growth factor (IGF), angiogenin (Ang), stromal cell-derived factor (SDF) and transforming growth factor (TGF) (2).

The heparan sulfate (HS) chains from endothelial cell proteoglycans interact with the major angiogenic factors, regulating blood vessels´ formation (3). In general, HS stabilizes the complex formation between angiogenic factors and the respective receptors (4). Furthermore, HS can also modulate the release of such angiogenic factors after the action of the enzyme heparanase or proteoglycan cleavage by proteases, forming a concentration gradient that may direct angiogenesis (5–7).

Heparin and HS exhibit structural similarities and share the same biosynthetic pathway. Both HS and heparin are formed by repeated disaccharide units of glucosamine and uronic acid (D-glucuronic acid or L-iduronic acid) residues linked by glycosidic α-intradisaccharide and β-interdisaccharide bonds (5, 7, 8).

Angiogenesis plays an essential role in physiological events such as placenta formation, cicatrization, and the menstrual cycle but is also essential in pathological processes, such as neoplasia (9). In addition, during tumorigenesis, the formation of neovascularization supplies nutrients and oxygen to tumor cells, allowing intense cell proliferation and enabling tumor metastases (9).

In 1971, it was hypothesized that the molecules involved in the angiogenic process could be a good target for antitumor therapy (10). Since then, several treatments have been investigated and developed (10).

In 2004, the US Food and Drug Administration approved using the first anti-VEGF-A monoclonal antibody, called bevacizumab, to treat colorectal cancer. Since then, anti-angiogenic therapy has shown temporary inhibition of tumor progression and increased patient survival. Moreover, the anti-angiogenic treatment combined with chemotherapeutic treatments proved to be more efficient, with a significant increase in survival rate than isolated chemotherapy (4, 10).

Previous work in our group has selected an HS-binding peptide using a phage display system. The biopanning assay was performed using the random peptide library expressed in the bacteriophage system to target the recombinant enzyme N-deacetylase N-sulfotransferase-1 (NDST1) involved in HS/heparin synthesis. The NDST1 enzyme removes the N-linked acetyl group at carbon 2 from glucosamine residue and adds a sulfate group at such a position (SO3-). In addition to selecting a peptide that binds to NDST, an HS ligand peptide was also selected since that HS is present at the catalytic domain of such recombinant enzyme (11).

Peptides have been used as a potential treatment in cancer. Arap and colleagues selected specific peptides that target to tumor vasculature of breast cancer, melanoma, and Kaposi’s sarcoma (12). The selection of specific peptides can be performed by screening using a phage display peptide library. George Smith first described filamentous phage as a random peptide library by fusing the peptide into capsid proteins.

This phage peptide library could be used to select a peptide that binds towards a specific target after rounds of binding assays. These peptides have lower production costs, good affinity, good tissue penetration, and little immune response (13).

Therefore, we were interested in evaluating the ligation specificity and the possible effect of the selected peptide in angiogenesis and tumor progression.

## Materials and Methods

### Animals

According to the University of California San Diego animal welfare guidelines, all animals were treated as described and approved by the UCSD Institutional Animal Care and Use Committee and comply with the ARRIVE guidelines (S12005 and S06008). Transgenic Tg[(Fli1: eGFP)] zebrafish were kindly provided by Dr. David Traver (UCSD). Zebrafish were maintained as previously described. C57BL/6 J mice were purchased from Jackson Laboratory, and mice were euthanized with CO_2_. All mice were maintained in a specific pathogen-free vivarium.

### Analysis of Tryptophan Emission

Tryptophan can be excited at wavelengths between 280-290 nm and emission at 350 nm. It is important to note that the emission spectrum of tryptophan can be altered depending on its conformation. Therefore, changes in the emission spectrum of tryptophan during the titration of some glycosaminoglycans (heparin, dermatan sulfate, and chondroitin sulfate) suggest an interaction between the glycosaminoglycan and the peptide. The synthetic peptide SADGARGWRGEKIGNGAAG (3 μM, Peptide 2.0, Chantilly, Virginia, USA) or the scramble peptide SADGAIENKWRGGRGGAAG (used as specificity control; 3 μM Peptide 2.0, Chantilly, Virginia, USA) was dissolved in phosphate-buffered saline, PBS (137 mM NaCl, 2.7 mM KCl, 10 mM Na_2_HPO_4_, 1.8 mM KH_2_PO_4_, pH 7.4). The peptide solution was filtered through a 0.22 μM sterilizing filter (Millipore, Billerica, Mass., USA), followed by collecting of the emission spectrum in a cuvette of quartz. Under constant stirring, each of the glycosaminoglycans were titrated separately, and the emission spectrum was collected by Fluorimeter (Shimadzu, Kyoto, Kyoto, Japan). Chondroitin sulfate from cartilage, and dermatan sulfate from bovine intestinal mucosa were obtained from Seikagaku (Seikagaku Kogyo Co, Tokyo, Japan) and and was obtained from Sigma-Aldrich, St. Louis, Missouri, USA.

### Circular Dichroism (CD)

Circular Dichroism spectra were recorded by Chirascan Plus (AppliedPhotophysics) using a quartz cuvette with a 0.1-mm optical path. The CD spectra (185-250nm) were collected with 12µM of peptide and 6.7 µg/mL of porcine heparin in sodium phosphate solution (10 mM). All spectra obtained represent the average of eight independent repetitions. Chemically modified heparins were used in the CD assays Heparin N-sulfated (HepNS); Heparin 2-O-Sulfated (Hep2S); Heparin 6-O-sulfated (Hep6S); Heparin 2-O-sulfated and 6-O-sulfated (Hep2S6S); Heparin 6-O-Sulfated and N-sulfated (Hep6SNS); Heparin 2-O-Sulfated and N-sulfated (Hep2SNS). The chemically modified heparins were graciously provided by Dr. Marcelo Lima (Universidade Federal de São Paulo). The modification was performed as described by Yates and co-workers (14). BestSel Software predicted the secondary structure and fold recognition.

### Cell Proliferation

Five thousand (5,000) human umbilical vein endothelial cells (HUVEC, ATCC) were plated in a 96-well plate with F-12 medium (ThermoFisher Scientific, Waltham, Mass., USA) containing 10% FBS, penicillin (100 U/ml), and streptomycin (100 μg/ml), 37°C, 5% CO_2_. HUVEC cells were treated with the selected HS-binding peptide at different concentrations (1 µM, 5 µM, 10 µM and 50 µM) for 18 hours. Proliferation analysis was performed using Cell Proliferation ELISA Kit, BrdU (Roche, Basel, Switzerland), following the manufacturer’s instructions. This assay was performed in triplicate.

### Capillary Formation

Matrigel (Becton, Dickinson-BD, Franklin Lakes, New Jersey, USA) was thawed at 4°C, and aliquots of 100 µl (10 mg/ml) applied to 96 well-plate (Corning, New York, New York, USA), which were maintained at 37°C in a humidified atmosphere with 2.5% CO_2_ for 16 h for gelation. Seven thousand (7,000) endothelial cells from human umbilical vein endothelial cells (HUVEC, ATCC) were plated in the 96-well plate coated with Matrigel (Becton, Dickinson-BD, Franklin Lakes, New Jersey, USA) in the presence of F-12 medium (Thermo Fisher Scientific, Waltham, Mass, USA), containing 10% fetal bovine serum, penicillin (100 U/ml) and streptomycin (100 µg/ml), 37°C, 5% CO_2_. The cells were treated in the presence or absence of HS-binding peptide (10 µM) for 18 hours. This assay was performed in quadruplicate. The tube formation was analyzed by microscopy (Zeiss, Oberkochen, Germany) 20x magnification. Three images were randomly taken in different areas and quantified by two different observers. The total length tubular structures on the Matrigel was measured and determined using image analysis software (AxioVision software, Carl Zeiss). The results are expressed as lumen length.

### Angiogenesis Assay Using Zebrafish Model

For analysis of angiogenesis zebrafish mutant Tg[(Fli1: eGFP)], embryos were cultured in E3 medium containing 1-phenyl 2-thiourea (PTU 0.003%) (Sigma-Aldrich, St. Louis, MO, USA) at 28°C. Different doses of HS-binding peptide or scrambled peptide were microinjected into the heart of 2-day post-fertilization (2 dpf) embryos. On the third day post-fertilization (3 dpf), the formation of subintestinal vessels was analyzed by confocal microscopy. The analysis was performed by confocal images (Nikon C1Si, Minato, Tokyo, Japan). For quantification, it was measured to the size of the subintestinal vessels using ImageJ software. Ten embryos were used in each experimental group, 10x magnification.

### Mouse Aortic Ring Assay

Two female C57BL mice (age 6 to 8 weeks) were euthanized with CO_2_ at the University of California San Diego; the experiments were approved and performed by relevant named guidelines and regulations. The aortas were removed with tweezers’ aid, adipose tissue, and other tissues detached to the aorta. Next, aortas were cut into 0.5 mm rings. The *ex vivo* aorta rings were incubated for 18 hours in an Opti-MEM culture medium (ThermoFisher Scientific, Waltham, Mass., USA) at 37°C, 5% CO_2_. Next, 60 μl of Matrigel (10 mg/mL) (Corning, New York, New York, USA) was added to the center of the 24-well plate, and the plate was incubated for 10 minutes at 37°C, 5% CO_2_. The aortic ring was placed on top of the Matrigel and incubated for 15 minutes at 37°C. Additionally, 50 μl of Matrigel (10 mg/mL) and incubated for an additional 30 minutes at 37°C. The culture of the aortas was maintained into Opti-MEM medium supplemented with 2.5% FBS, penicillin (100 U/mL), streptomycin (100 μg/mL), 50 ng/mL VEGF-A and 20 ng/mL FGF-2. The aortas were incubated for 7 days, 37°C, 5% CO_2_, with the treatment of the HS-binding peptide (10 μM and 100 μM) or scramble peptide (10 μM). Approximately 50% of the culture medium was changed every 4 days. The peptides and growth factors (VEGF-A, FGF-2) were replenished every 2 days. The aortic rings were analyzed by microscopy (Zeiss). This assay was performed in quadruplicate using 10x magnification.

### Cell Index of HUVEC Cells in the Presence of VEGF-A and FGF-2

HUVEC (ATCC) cells (1,500 cells) were cultured in a 96-well plate containing type 1 collagen from rat tail (Sigma-Aldrich, St. Louis, Missouri, USA). First, the culture plates were pretreated with type 1 collagen (10 μg/ml, 1-hour incubation, 37°C). Then, cells were plated in Opti-MEM culture medium (ThermoFisher Scientific, Waltham, Massachusetts, USA), containing 2% FBS, penicillin (100 U/ml), streptomycin (100 μg/ml), 50 ng/ml VEGF-A or 20 ng/ml FGF-2, in the presence of 10 μM of the HS-binding peptide. During 50 hours, the cell index number was determined using xCELLigence (ACEA Bioscience, San Diego, California, USA). The cell index is directly related to the rate of cell proliferation and cell viability. The experiment was performed in triplicate.

### 3D Culture

PDX (patient-derived tumor xenograft) cells from a patient with a triple-negative breast tumor were used. PDX cells were kindly provided by CROWN Bioscience (San Diego, California, USA). Briefly, the tumor was collected from the patient and injected into a NOD/SCID mouse. After 21 days, the tumor was resected and digested with collagenase for tissue dissociation. Approximately 2,500 PDX cells in 50 μl DMEM/F-12 medium (ThermoFisher Scientific, Waltham, Mass., USA) were supplemented with 10% fetal bovine serum, 100 U/ml penicillin, 100 μg/mL streptomycin, and glutamine/gentamicin solution (Sigma-Aldrich, St. Louis, Missouri, USA). The cells were plated on a 96-well plate (Corning, New York, New York, USA) coated with 50 μl of Matrigel (10 mg/mL) (Corning, New York, New York, USA). Next, culture medium DMEM/F12 containing 5% of Matrigel was added on top of cell culture. The cells were treated with 10 μM or 100 μM of the HS-binding peptide and cultured for 14 days, 37°C, 5% CO_2_ to obtain the spheres (3D cultures). Exchange of the culture medium and replacement of HS-binding peptide was performed every three days. Cell proliferation and viability analysis were determined by the size of the colonies (area). Measurements were obtained using a Nikon microscope using 10X magnification (Nikon, Minato, Tokyo, Japan). The assay was performed in triplicate.

### Effect of HS-Binding Peptide on Tumor Progression

The chorion of the zebrafish embryos Tg[(Fli1: eGFP)], with 1-day post-fertilization (1 dpf), were manually removed with tweezers, the was anesthetized with tricaine, and immobilized. The experiments were performed following relevant named guidelines and regulations in the University of California San Diego. After immobilization, the embryos were microinjected with 150 cells. The same PDX cells (triple-negative breast cancer) obtained in the 3D culture described previously were used for the xenograft assay in zebrafish. Embryos were incubated at 35.5°C for 18 hours and then treated with the injection of 10 μM of the HS-binding peptide. Treatment analysis was completed at 3 dpf. Microinjection was performed in the yolk sac of the animal. PDX cells were first transduced with lentivirus to express a red fluorescent protein (RFP) for fluorescent labeling of tumor cells. The translucent lentivirus cells were selected using 2 μg/ml puromycin for 5 days. The cells exhibiting the greatest expression of the red fluorescent protein were selected by cell sorting BD Influx (Becton, Dickinson-BD). Analysis of the HS-binding peptide effect on tumor progression was performed by 10X magnification confocal microscopy (Nikon C1Si, Minato, Tokyo, Japan), and the amount of red tumor fluorescence was measured by ImageJ software. Also, the survival of the animals was analyzed. The tests were performed with ten zebrafish embryos per group.

## Results

The HS binding peptide RGWRGEKIGN was selected by the phage display system published by our group. The synthetic HS-binding peptide used for the experimental assays presents flanked amino acid residues displayed at the pIII capsid protein, where the peptide was expressed in the bacteriophage library. The HS-binding peptide’s affinity was previously analyzed by HS/heparin interaction assays, as described in the article already published by Gesteira et al. (11).

### Characterization of the HS-Binding Peptide

**Figure 1:**
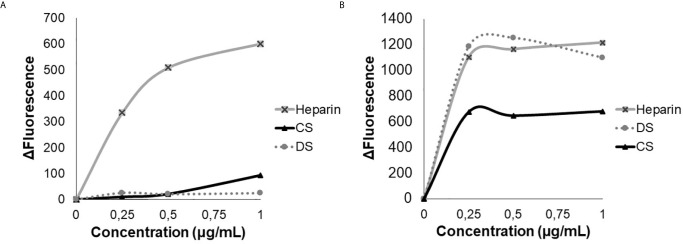
Analysis of tryptophan emission. Heparin, dermatan sulfate (DS) and chondroitin sulfate (CS), were titrated in the presence of 3 µM of HS-binding peptide **(A)** or in the presence of 3 µM of scrambled peptide **(B)**. Excitation 280 nm, emission 350 nm. The HS- binding peptide interacts only with heparin.

The HS-binding peptide interaction with heparin was performed by the emission spectrum of tryptophan (W), showing that HS-binding peptide interacted exclusively with heparin, while the scrambled peptide (SADGAIENKWRGGRGGAAG) interacted with heparin, dermatan sulfate (DS), and chondroitin sulfate (CS). The results showed that the scrambled peptide has lower specificity since it binds with other glycosaminoglycans and presents a higher affinity to heparin than the HS-binding peptide. Such results show that the amino acid sequence is important for the HS/heparin-binding specificity (**Figure 1**).

Considering heparin as an analog of heparan sulfate, we used chemically modified heparins to evaluate the HS-binding peptide´s affinity by dichroism analysis. In addition, nuclear magnetic resonance (NMR) was used to characterize each modified heparin (data are not shown). It is noteworthy that the sulfation profile at the C3 position of glucosamine residues was not analyzed; therefore, we cannot determine whether or not there is sulfation at such position in the different modified heparin molecules used in the present study.

The synthetic peptide SADGARGWRGEKIGNGAAG was analyzed by circular dichroism. The peptide presents 41.7% of β-sheet antiparallel conformation, 14.3% turn, 44.1% other conformation. Additionally, 25.9% of β-sheet antiparallel conformation is right-twisted.

**Figure 2:**
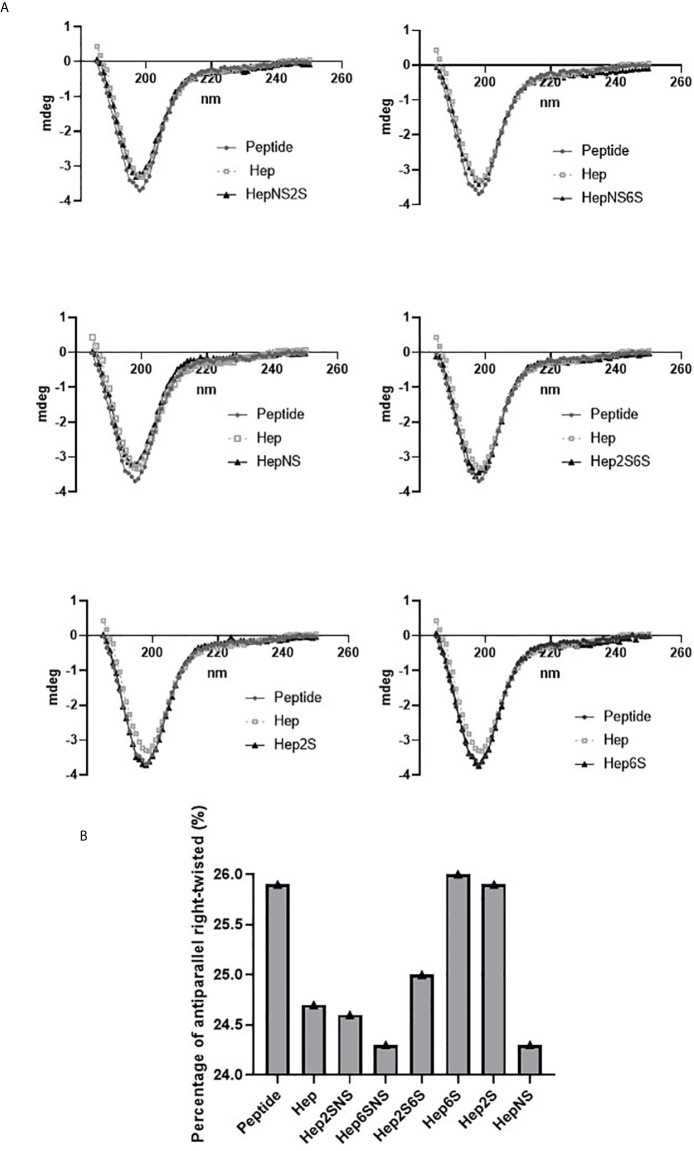
A spectrum obtained by circular dichroism. Circular dichroism was performed to determine the peptide interaction with chemically modified heparins, demonstrating an alteration in the secondary structure of this peptide. Porcine heparin (Hep), Heparin 2-O-sulfated and N-sulfated heparin (Hep2SNS), Heparin 6-O-sulfated and N-sulfated (Hep6SNS), Heparin 2-O-sulfated and 6-O-sulfated (Hep2S6S), Heparin 6-sulfated (Hep6S), Heparin 2-sulfated (Hep2S), and Heparin N-sulfated (HepNS). The heparins that showed the greatest interaction with the peptide were the molecules containing N-sulfation. The assay was performed in the presence of 12 µM of the HS binding peptide and 6.7 µg/ml of each heparin in a 10 mM sodium phosphate solution. Control, 12µM of peptide in 10mM sodium phosphate solution. **(A)** The lines represent the repeat averages of eight different experimental readings. **(B)** The percentage of right-twisted antiparallel beta-sheet was analyzed. A reduction from the right-twisted shape to the left-twisted or relaxed shape was observed specifically with N-sulfated heparins.

The dichroism analysis shows that the HS-binding peptide has a higher affinity for heparin N-sulfated (HepNS) Heparin 2-O-sulfated and N-sulfated (Hep2SNS); heparin 6-O-sulfated and N-sulfated (Hep6SNS) and heparin N-sulfated (HepNS) and lower affinity for N-acetylated heparins, suggesting specific interaction with the N-sulfation pattern (**Figure 2A**).

The HS-binding peptide interaction with modified heparins decreased the right-twisted conformation percentage since the interaction with heparin replaced the right-twisted conformation with left-twisted or relaxed β-sheet antiparallel. (**Figure 2B**).

Furthermore, the secondary structure analysis predicted by Best Software detected a similarity between HS binding peptide with Taiwan snake cardiotoxin A3 named PDB 2BHI, which is a polypeptide containing a heparin-binding domain corroborating our results (15).

### Effect of HS-Binding Peptide in Angiogenesis

**Figure 3:**
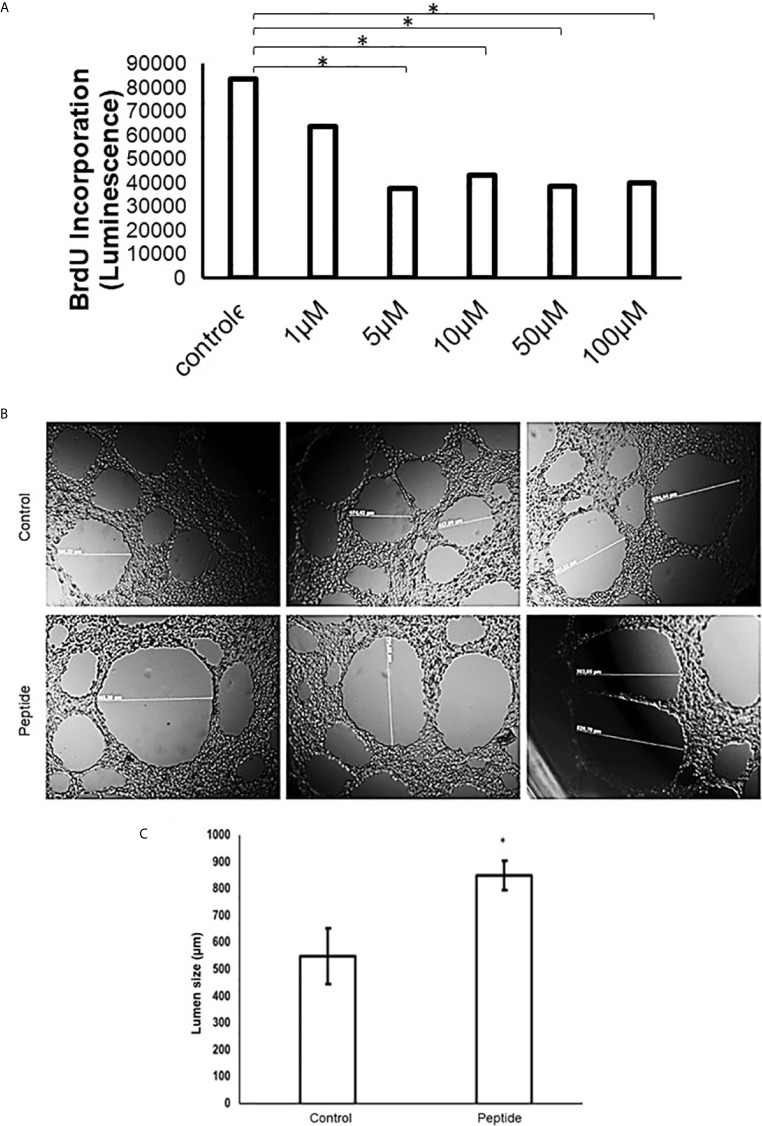
Angiogenesis *in vitro* analysis. **(A)** Cell proliferation assay in the presence of HS-binding peptide. Proliferation assays were performed using BrdU as described in Methods. HUVEC cells were treated with the HS-binding peptide at different doses for 18 hours. After treatment, BrdU was added into the cell culture and luminescence was analyzed. Assay performed in triplicate, the bar represents average and lines represent standard deviation. The proliferation is inhibited from 1 µM of HS-binding peptide and reaches maximum inhibition at 5 µM. *p < 0.05 (Kruskall-Wallis). **(B)** Tubular formation with endothelial cells. Control, Cells remain in the absence of treatment. Peptide, Cells were treated with the HS-binding peptide (10 µM) for 18 hours. The tubular formation was analyzed by microscopy 20x magnification. **(C)** Lumen size was measured, the bar represents average and lines represent standard deviation, *p < 0.05 (Kruskall-Wallis). Control, cells remain in the absence of treatment. Peptide, cells were treated with HS-binding peptide (10µM) for 18hours. It was verified that peptide was capable to inhibit the tubular formation of endothelial cells.

The *in vitro* proliferating assay using BrdU shows HS-binding peptide could inhibit the proliferation of human endothelial umbilical cord cells (HUVEC) at doses from 1 to 100 μM of the, reaching the maximum inhibition the concentration of 5 μM (**Figure 3**).

Also, there was a significant alteration in the tube formation when HUVEC cells were treated with 10 µM of HS-binding peptide for 18 hours. The capillaries had a higher diameter, or the lumen formation was abrogated formed compared to non-treated cells, as shown in **Figure 3**.

**Figure 4:**
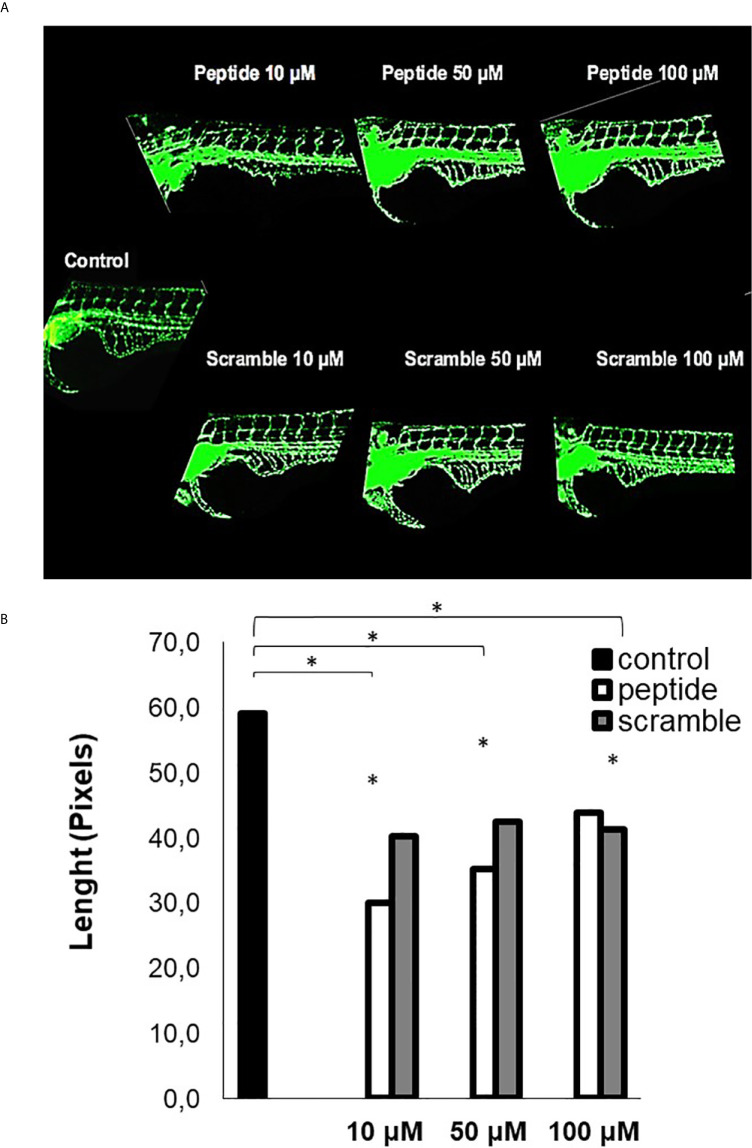
Analysis of angiogenesis in *vivo*. This assay was performed with Tg [Fli1: ​​eGFP] embryos (10 embryos per group). **(A)** the image of zebrafish embryos. Control, untreated embryos. Peptide, embryos treated with the HS-binding peptide. Scramble, embryos treated with scrambled peptide. Assays were performed with zebrafish embryos 2 days after fertilization (2 dpf). After 24 hours of treatment, the animals were analyzed by confocal microscopy and the images obtained in 10X magnification. Subintestinal vessels were analyzed. **(B)** the quantification of the subintestinal vessels was evaluated using ImageJ software. The values ​​express the mean and standard deviation in each group. *p < 0.05 (ANOVA). The HS-binding peptide was capable of inhibiting angiogenesis of sub intestinal vessels.

The assay using such peptide was performed with transgenic zebrafish embryos Tg (Fli1:eGFP), that express a green fluorescent protein (GFP) in blood vessels, which allowed us to investigate some effect of the HS-binding peptide *in vivo*. **Figure 4** demonstrates that HS-binding peptide (10 μM) inhibits the formation of sub-intestinal blood vessels in zebrafish embryos Tg(Fli1:eGFP), confirming the anti-angiogenic activity of such peptide obtained by *in vitro* assays using HUVEC cell line. However, there was no benefit for using a higher dose of the HS-binding peptide (50-100 μM), confirming *in vitro* experiments described previously.

**Figure 5:**
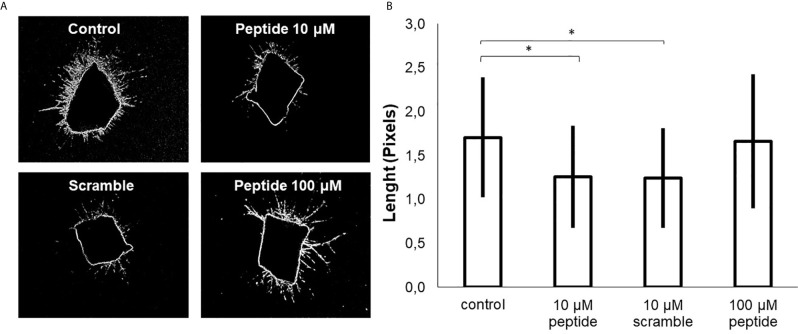
*Ex vivo* angiogenesis assay. Sixteen rings of mouse aortas were collected and cultured in culture plates containing Matrigel for seven days, **(A)** aorta ring cultured in the absence of peptide (control); aorta ring cultured in the presence of HS-binding peptide (10 µM or 100 µM) and aorta ring cultured in the presence of scrambled peptide (10 µM). **(B)** The area of ​​the formed blood vessels was analyzed using ImageJ software. The values represent means and standard deviations the area reached by the blood vessels formed from the aorta. *p < 0.05 (ANOVA). The HS-binding peptide decreased blood formation from the aorta.

Aortas were collected from two C57BL/6 mice, sectioned in rings, and cultured in Matrigel for seven days to evaluate new blood vessel formation. HS-binding peptide decreased the number and the size of blood vessels in sectioned aorta rings (**Figure 5**). Thus, this *ex vivo* experimental model reinforces the inhibitory effect of the HS-binding peptide. Furthermore, the ex vivo assay also confirmed that increasing the HS-binding peptide doses did not intensify the anti-angiogenic effect.

### Possible Mechanisms Involved With Angiogenesis Inhibition

**Figure 6:**
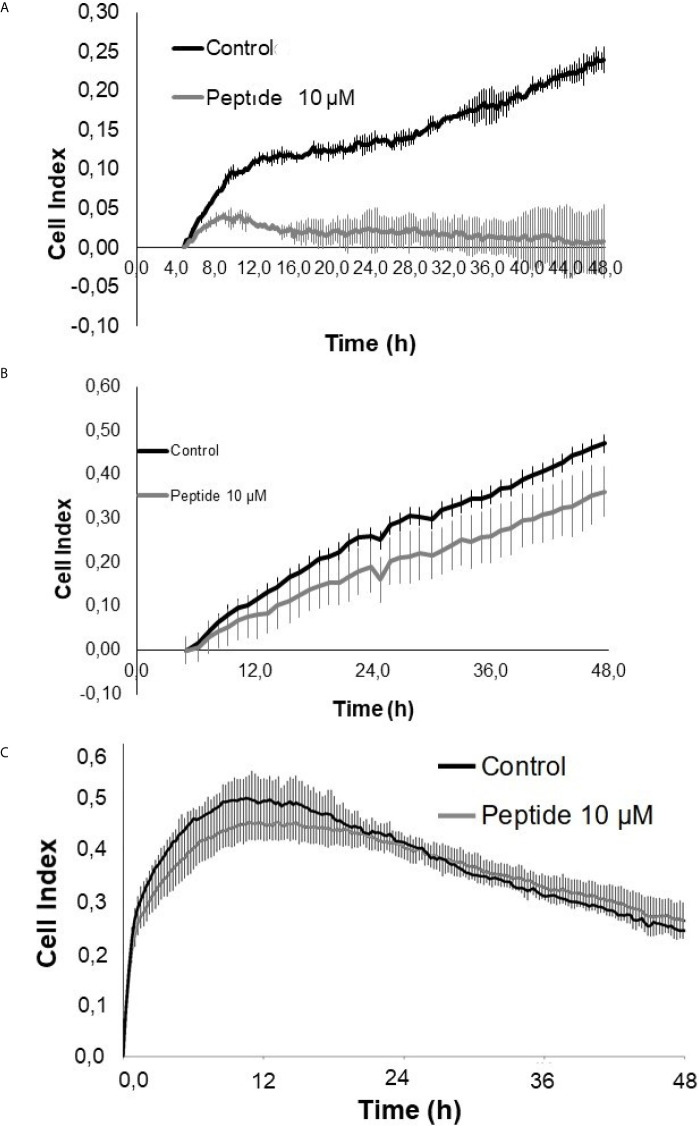
Cell proliferation/viability assay in the presence of growth factors. HUVEC cells were cultured for 50 hours on collagen type 1 in medium containing 2% fetal bovine serum (FBS) and 20 ng/mL FGF-2 or 50 ng/mL VEGF-A, in the presence or absence of the HS-binding peptide. **(A)** VEGF-A + 2% SFB. **(B)** FGF-2 + 2% SFB. **(C)** 2% SFB. Control, HUVEC cells in the absence of treatment. Peptide, HUVEC cells with HS-binding peptide treatment (10 µM). Assay performed in triplicate. The values indicate the mean and standard deviation of the cell index (index proportional to the number of cells adhered to the plate). Peptide inhibited FGF-2 proliferation. Each point of FGF-2 assay was statically significant p<0.05 (Kruskall-Wallis) while the decrease of proliferation/viability with VEGF-A was not significant.

Cell index analysis of HUVEC cells in the presence of VEGF-A or FGF-2 was performed using xCELLigence equipment (ACEA Bioscience, San Diego, California, USA). The results showed that the HS-binding peptide inhibited the rate of endothelial cells´ proliferation and viability in the presence of FGF-2 (**Figure 6A**). However, the proliferation and viability of HUVEC cells were not significant altered by the HS-binding peptide in the presence of VEGF-A (**Figure 6B**).

A control assay was performed exclusively in the presence of fetal bovine serum (FBS) to confirm that the enhancement in the proliferation and viability of HUVEC cells was related to FGF-2 and had not been affected by growth factors that were present in the culture medium, which was supplemented by FBS (**Figure 6C**).

The data suggest that decreased HUVEC cell proliferation after treatment of the HS-binding peptide may be mediated by inhibiting the interaction between HS and FGF-2.

### Effect of HS-Binding Peptide on Tumor Progression

**Figure 7:**
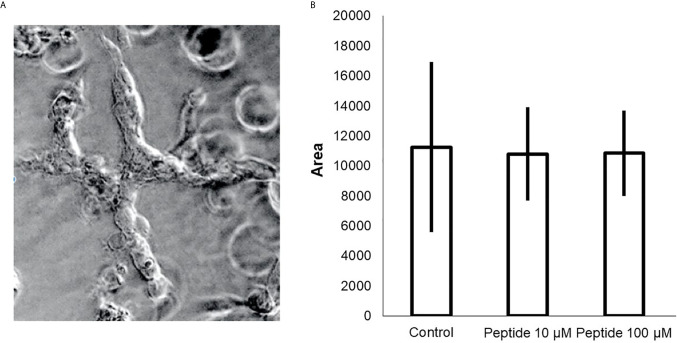
Effect of HS-binding peptide in the 3D culture (spheroid) triple-negative breast cancer. Patient-derived xenotransplant cells (PDX cells) were obtained from tissue collected of a triple negative breast cancer patient. **(A)** The cells cultured for 14 days. The exchange of culture medium was performed every three days. **(B)** Cell proliferation and viability analysis were determined by the size of the colonies (area). Measurements were obtained using a Nikon microscope using 10X magnification (Nikon, Minato, Tokyo, Japan). The assay was performed in triplicate. Peptide did not affect triple-negative cells, p>0.05 (Kruskall-Wallis).

Finally, we sought to determine whether the HS-binding peptide could block tumor growth. A tissue biopsy collected from resection of a triple-negative breast cancer patient was cultured in Matrigel to obtain a 3D culture. Patient-derived xenotransplant cells (PDX cells) were maintained for 14 days in a culture medium in the presence or absence of the HS-binding peptide. There was no change in the growth of the 3D culture of PDX colonies after HS binding-peptide treatment, as shown in **Figure 7**.

**Figure 8:**
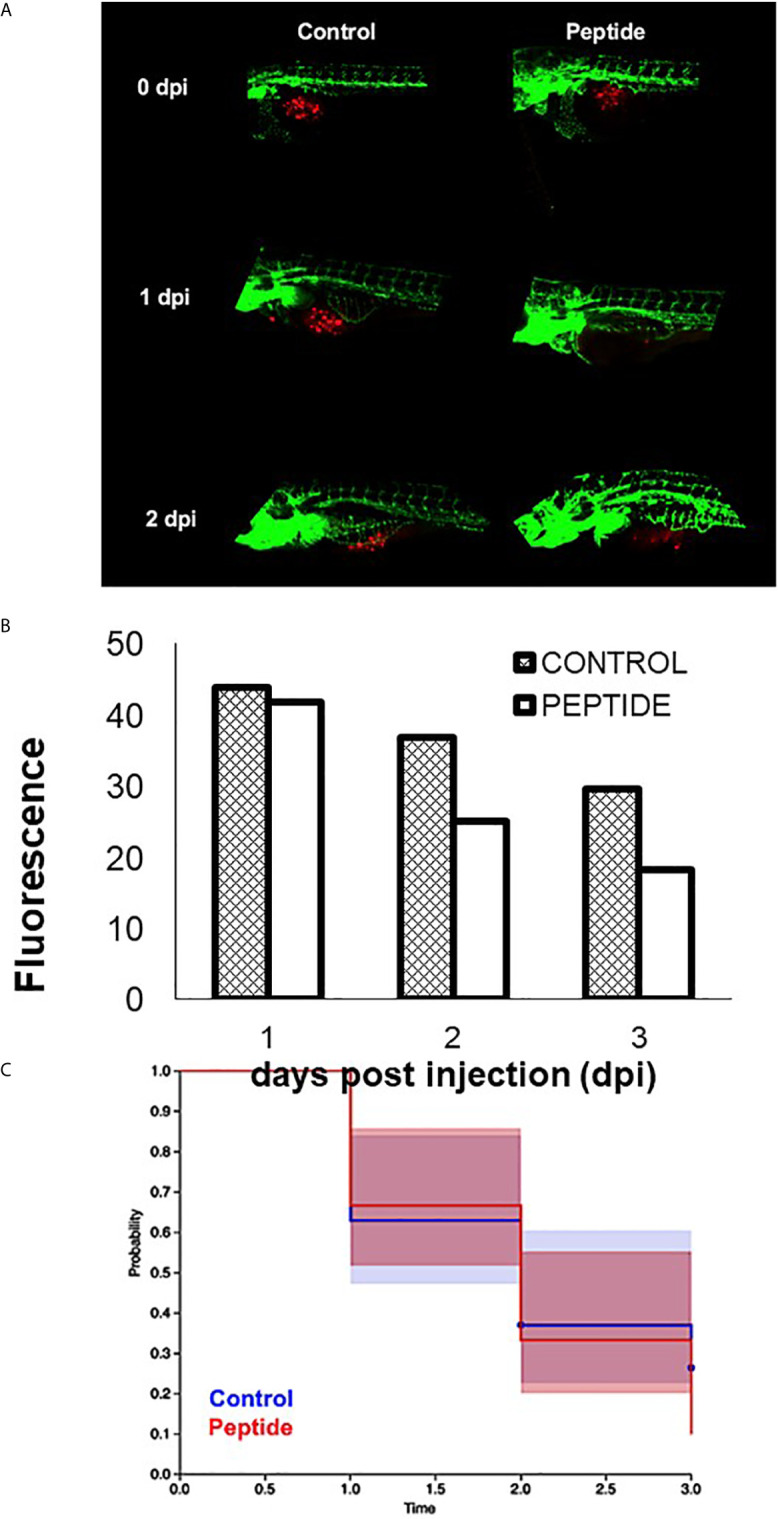
Effect of HS-binding peptide on tumor progression. Approximately 150 PDX cells obtained by surgical resection of a patient with triple-negative breast cancer were labeled with red fluorescent protein (RFP) and injected into the zebrafish embryo yolk sac after 1 day of fertilization (1 dpf). Embryos were incubated for 18 hours, 35.5°C. Peptide, animals were treated with 10 µM HS-binding peptide. Control, animals were not treated. Green; green fluorescent protein (GFP) labeled blood vessels. Red; red fluorescent protein (RFP) labeled tumor cells. **(A)** Representative images of the control group and the treated group. **(B)** Quantification of tumor fluorescence (tumor size). The bar represent the average of red fluorescence in zebrafish embryos. **(C)** Values express percent survival of animals; red line (10 μM of peptide treatment); blue line (animals in the absence of treatment). Each group contains 10 zebrafish embryos. Peptide decreased the number of tumoral cells and increased survival.

However, when triple-negative PDX cells were injected in zebrafish embryos Tg (Fli1: eGFP) and subsequently treated with HS-binding peptide, the tumor size decreased, and there was also an increase in the survival rate of the zebrafish embryos (**Figure 8**), suggesting an antitumor efficacy of HS-binding peptide.

We hypothesize that the decrease in tumor growth promoted by the HS-binding peptide in the *in vivo* assay is possibly due to inhibition of angiogenesis since the peptide does not appear to alter the viability or the proliferation of triple-negative breast tumor cells.

## Discussion

Breast tumors are classified according to the presence of some plasma membrane receptors, such as estrogen receptors (ER), progesterone receptors (PR), and human epidermal growth factor type 2 receptor (HER-2). Triple-negative breast cancer has low or absent ER, PR, and HER-2 expression. Consequently, hormone treatment or treatment with the anti-HER-2 monoclonal antibody does not have efficacy in these patients (16). Also, triple-negative breast tumor has a higher growth rate compared to the other types of breast tumors. Therefore, new treatment alternatives are important for tumors such as triple-negative breast cancer.

Tumor progression steps include tumor cells´ proliferation, the formation of new blood vessels, and metastasis. The proliferation of the tumor cells and metastatic events is dependent on the neovascularization that nourishes and carries oxygen to the tumor, besides allowing the extravasation of tumor cells. Thus, angiogenesis favors tumor progression and the invasion of tumor cells (16).

The angiogenic process involves cross-talk between tumor cells, stromal cells, and endothelial cells. Tumor and stromal cells appear to activate proliferation, migration and modify endothelial cell phenotype due to the release of soluble factors, mainly VEGF and FGF-2 (4, 9).

The heparan sulfate proteoglycans present in the plasma membrane modulate various cellular responses, including cell proliferation, migration and adhesion, apoptosis, inflammatory response, and angiogenesis (7). It is known that the structural modifications of HS and heparin molecules promote significant changes in the interactions of a variety of compounds. The degree of sulfation and the distribution and conformation of D-glucuronic acid and L-iduronic acid residues interfere with HS/heparin molecule conformation, affecting the interaction of HS with other molecules (5).

It is important to emphasize that the HS-binding peptide was selected by Phage Display using as target endogenous heparan sulfate present at the active site of the recombinant purified enzyme N-deacetylase-N-sulfotransferase-1 (NDST-1) (11).

Moreover, HS-binding peptide has structural similarity with the polypeptide Taiwan snake cardiotoxin A3 (PDB 2BHI), which presents a heparin-binding domain results (15).

The HS-binding peptide has a majority antiparallel beta-sheet conformation that is characteristic of peptides with a heparin-binding site (15, 17).

The present results demonstrate that HS-binding peptide interacts preferentially with N-sulfated heparin. It is well known that N-sulfation and 2-O-sulfation are required for HS binding with FGF-2, while sulfation at the carbon 6 of glucosamine residues is specifically important for FGFR-1 binding (18–24). Our results corroborate these previous studies confirming that HS-binding peptide interacts with N-sulfated and 2-O-sulfated heparin, representing FGF-specific binding domains in the HS molecule, indicating that such an anti-angiogenic effect peptide might be controlled by the specific interaction between FGF-2 and such HS domains.

The involvement of HS proteoglycans (HSPG) in the formation of new blood vessels is well-known. Studies with zebrafish have shown that the decrease of HSPG, syndecan-2, reduces the migration of endothelial cells during the formation of new capillaries during embryonic development and indicates that such inhibition is modulated by VEGF (25). Moreover, some data in the literature demonstrate that a VEGF analogous peptide interacts with HS chains, promotes inhibition of endothelial cell migration, and decreases tumor size in the mouse model (26). Consequently, HS involvement in forming new blood vessels is obvious, which is essential for carcinogenesis.

In this context, the HS-binding peptide indicates a therapeutic potential to inhibit angiogenesis. Moreover, the data showed that HS-binding peptide was able to decrease tumor growth and increase survival of zebrafish embryos, likely by promoting the formation of fewer new blood vessels and not by the direct effect on the tumor cell, since *in vitro* assays showed that HS-binding peptide did not alter the viability and the proliferation of triple-negative breast tumor cells. Therefore, HS-binding peptide may represent a potential adjuvant treatment for triple-negative breast tumors, known to be poorly responsive to conventional therapy such as chemotherapy.

The selected HS-binding peptide interacts with a specific structure of heparan sulfate and inhibits tumor growth of triple-negative breast cancer cells, possibly interfering with the proliferation signaling modulated by FGF-2 and consequently decreasing angiogenesis.

Despite molecular mechanisms that need to be further investigated, the set of results obtained in the present study highlights the potential use of the HS-binding peptide as an angiogenesis inhibitor and might be useful in combination with other antitumor drugs in cancer therapy.

## Data Availability Statement

The raw data supporting the conclusions of this article will be made available by the authors, without undue reservation.

## Ethics Statement

According to the University of California San Diego animal welfare guidelines, all animals were treated as described and approved by the UCSD Institutional Animal Care and Use Committee and in compliance with the ARRIVE guidelines (S12005 and S06008). Transgenic Tg[(Fli1: eGFP)] zebrafish were kindly provided by Dr. David Traver (UCSD). Zebrafish were maintained as previously described. C57BL/6 J mice were purchased from Jackson Laboratory, and mice were euthanized with CO2. All mice were maintained in a specific pathogen-free vivarium.

## Author Contributions

CM, contributions to the conception; the acquisition and analysis; interpretation of data; have drafted the work or substantively revised it. HW, contributions to the conception; acquisition and analysis; FK, the acquisition and analysis; SJ, the acquisition and analysis; MMCZ, the acquisition and analysis; NHB, have drafted the work or substantively revised; KRL, have drafted the work or substantively revised; PMAS, contribution to the conception, interpretation of data, have drafted the work or substantively revised. All authors contributed to the article and approved the submitted version.

## Funding

This work was supported by FAPESP (process number 2016/01357-8 and 2019/03024-4), CNPQ and CAPES.

## Conflict of Interest

The authors declare that the research was conducted in the absence of any commercial or financial relationships that could be construed as a potential conflict of interest.

## Publisher’s Note

All claims expressed in this article are solely those of the authors and do not necessarily represent those of their affiliated organizations, or those of the publisher, the editors and the reviewers. Any product that may be evaluated in this article, or claim that may be made by its manufacturer, is not guaranteed or endorsed by the publisher.
